# ETS1, ELK1, and ETV4 Transcription Factors Regulate Angiopoietin-1 Signaling and the Angiogenic Response in Endothelial Cells

**DOI:** 10.3389/fphys.2021.683651

**Published:** 2021-07-26

**Authors:** Sharon Harel, Veronica Sanchez, Alaa Moamer, Javier E. Sanchez-Galan, Mohammad N. Abid Hussein, Dominique Mayaki, Mathieu Blanchette, Sabah N. A. Hussain

**Affiliations:** ^1^Translational Research in Respiratory Diseases Program, Research Institute of the McGill University Health Centre, Montreal, QC, Canada; ^2^Department of Critical Care, McGill University Health Centre, Montreal, QC, Canada; ^3^Meakins-Christie Laboratories, Department of Medicine, McGill University, Montreal, QC, Canada; ^4^School of Computer Science, McGill Centre for Bioinformatics, McGill University, Montreal, QC, Canada; ^5^School of Engineering and Technology (SET), Aldar University College, Dubai, United Arab Emirates

**Keywords:** angiogenesis, angiopoietins, endothelial cells, cell signaling, transcription factors, cell migration, proliferation, Tie-2 receptors

## Abstract

**Background:**

Angiopoietin-1 (Ang-1) is the main ligand of Tie-2 receptors. It promotes endothelial cell (EC) survival, migration, and differentiation. Little is known about the transcription factors (TFs) in ECs that are downstream from Tie-2 receptors.

**Objective:**

The main objective of this study is to identify the roles of the ETS family of TFs in Ang-1 signaling and the angiogenic response.

**Methods:**

*In silico* enrichment analyses that were designed to predict TF binding sites of the promotors of eighty-six Ang-1-upregulated genes showed significant enrichment of ETS1, ELK1, and ETV4 binding sites in ECs. Human umbilical vein endothelial cells (HUVECs) were exposed for different time periods to recombinant Ang-1 protein and mRNA levels of ETS1, ELK1, and ETV4 were measured with qPCR and intracellular localization of these transcription factors was assessed with immunofluorescence. Electrophoretic mobility shift assays and reporter assays were used to assess activation of ETS1, ELK1, and ETV4 in response to Ang-1 exposure. The functional roles of these TFs in Ang-1-induced endothelial cell survival, migration, differentiation, and gene regulation were evaluated by using a loss-of-function approach (transfection with siRNA oligos).

**Results:**

Ang-1 exposure increased ETS1 mRNA levels but had no effect on ELK1 or ETV4 levels. Immunostaining revealed that in control ECs, ETS1 has nuclear localization whereas ELK1 and ETV4 are localized to the nucleus and the cytosol. Ang-1 exposure increased nuclear intensity of ETS1 protein and enhanced nuclear mobilization of ELK1 and ETV4. Selective siRNA knockdown of ETS1, ELK1, and ETV4 showed that these TFs are required for Ang-1-induced EC survival and differentiation of cells, while ETS1 and ETV4 are required for Ang-1-induced EC migration. Moreover, ETS1, ELK1, and ETV4 knockdown inhibited Ang-1-induced upregulation of thirteen, eight, and nine pro-angiogenesis genes, respectively.

**Conclusion:**

We conclude that ETS1, ELK1, and ETV4 transcription factors play significant angiogenic roles in Ang-1 signaling in ECs.

## Introduction

The TEK receptor tyrosine kinase (Tie-2) and its ligand angiopoietin-1 (ANPGT1, Ang-1) have emerged as important regulators of angiogenesis, both in adults and in embryos. In adult mice, the Ang-1/Tie-2 receptor pathway stimulates vascular remodeling, vascular enlargement, wound healing, and lymphangiogenesis (Brindle et al., 2006). It also inhibits endothelial cell (EC) apoptosis and stimulates proliferation, migration, and differentiation of these cells (Kolizek et al., 1998; Witzenbichler et al., 1998; Kim et al., 2000; Brindle et al., 2006). Ang-1 stimulates the ERK1/2, p38, SAPK/JNK, PI3 kinase/AKT, and mTOR pathways (Kim et al., 2000; Harfouche et al., 2003; Abdel-Malak et al., 2007). Despite the importance of the Ang-1/Tie-2 receptor pathway to vascular homeostasis and angiogenesis, relatively little progress has been made toward the identification of transcription factors (TFs) that mediate its angiogenic responses. Our group has reported that Ang-1 triggers transient induction of early growth response-1 (EGR1), a TF that contributes to Ang-1-induced EC proliferation and migration (Abdel-Malak et al., 2008a). Other groups have shown that activator protein-1 (AP-1), which mediates the production of interleukin-8, and KLF2, which is involved in vascular quiescence and mediates anti-inflammatory effects of Ang-1 (Abdel-Malak et al., 2008b; Sako et al., 2008), are activated by the Ang-1/Tie-2 pathway in ECs, but the signaling and angiogenic contributions of other TFs associated with the pathway remain unknown.

In mammalian cells, the ETS (E-twenty-six or E26 transformation-specific) family of TFs consists of over 25 members that share a conserved DNA binding (ETS) domain that consists of 85 amino acids. They are important regulators of various cellular functions; including proliferation, migration, differentiation, inflammation, apoptosis, angiogenesis, and the cell cycle (Iwasaka et al., 1996; Nakano et al., 2000; Petrovic et al., 2003; Shimizu et al., 2004; Birdsey et al., 2008). In ECs, members of the ETS family are upregulated by pro-angiogenic signaling proteins like vascular endothelial growth factor (VEGF) and fibroblast growth factors (FGFs) and synergistically contribute to their effects on angiogenesis (Forough et al., 2006; Heo et al., 2010). On activation, they bind promoter regions of several pro-angiogenesis genes, including Ang-2, Tie-1, Tie-2, FLT1 (VEGF receptor), EGR1, and von Willebrand Factor (VWF) (Randi et al., 2009).

To our knowledge, two members of the ETS family of TFs that has been identified as being directly activated by the Ang-1/Tie-2 pathway including NERF2 and ERG. Christensen et al. (2002) reported that exposure of ECs to hypoxia resulted in upregulation of Tie-2 and NERF2 and that Ang-1 directly upregulated NERF2 expression in quiescent cells. They concluded that Ang-1 regulates NERF2 and Tie-2 expression in hypoxic ECs. More recently, Shah and colleagues (Shah et al., 2017) reported that the transcription factor ERG controls the balance between Notch ligands by repressing Jagged 1 expression and upregulating delta-like ligand 4 (DII4). They also found that ERG mediates Ang-1-dependent regulation of Notch ligands and is required for the stabilizing effects of Ang-1. Whether other members of the ETS family are also activated by Ang-1 remains unknown. The primary aim of this study is to identify ETS TFs that are activated by the Ang-1/Tie-2 pathway and to determine whether they contribute to Ang-1-induced angiogenesis in ECs.

## Materials and Methods

### Materials

Antibody for E26 transformation-specific sequence-1 (ETS1) detection was purchased from Novus Biologicals (Centennial, CO). Antibodies for ETS like-1 protein (ELK1), ETS variant transcription factor 4 (ETV4, also known as PEA3), and β-Tubulin were purchased from Santa Cruz Biotechnology (Santa Cruz, CA) and Novus Biologicals (Centennial, CO). Recombinant Ang-1 protein was purchased from R&D Systems Inc. (Minneapolis, MN). Human umbilical vein endothelial cells (HUVECs) were purchased from GlycoTech (Gaithersburg, MD).

### Cell Culture

HUVECs were used between passages 4–7. Cells were grown in complete MCDB131^®^ medium (Life Technologies, Rockville, MD) supplemented with 20% fetal bovine serum (FBS), endothelial cell growth supplement, 2 mmol/L glutamine, heparin, and gentamicin and incubated at 37°C and 5% CO_2_.

### Regulation of TF Expression

HUVECs were maintained in basic MCDB 131 medium (2% FBS) for 6 h. Cells were then exposed to PBS or Ang-1 (300 ng/ml) for 30 min, 1 h, or 3 h in the absence and presence of selective pharmacological inhibitors of ERK1/2 (U0126, 20 μM), p38 (BIRB796, 10 nM), SAPK/JNK (SP600125, 20 μM), PI-3 kinase (wortmannin, 100 nM); and mTOR (rapamycin, 50 ng/ml) pathways. Our group and other investigators have verified the selectivity of these inhibitors in ECs and other cells (Nwaozuzu et al., 2003; Kuma et al., 2005; Abdel-Malak et al., 2007, 2008a, b; Bolon et al., 2007; Echavarria and Hussain, 2013). Total RNA was then extracted using a PureLink^®^ RNA Mini Kit (Life Technologies). mRNA levels of TF and other genes were detected using real time qPCR with specific primers, SYBR^®^ green, and a 7500 Real-Time PCR System (Supplementary Table 1). GAPDH and β-ACTIN were used as control genes. All experiments were performed in triplicate. Relative mRNA expression was determined using the C_*T*_ method (2^–Δ^ ^Δ^ ^*CT*^), as previously described (Abdel-Malak et al., 2007). To determine absolute copy numbers of TFs and β-ACTIN mRNA transcripts, standard curves that related cycle threshold (C_*T*_) values to copy numbers were established, as previously described (Mofarrahi and Hussain, 2011). Copy numbers of individual TFs were then normalized per copies of β-ACTIN.

### Immunoblotting

HUVECs were maintained in basic MCDB 131 medium (2% FBS) for 6 h. Cells were then exposed to PBS or Ang-1 (300 ng/ml) for 1 h or 3 h. Cells were lysed using RIPA buffer (Santa Cruz Biotechnology, Dallas, TX). Denatured proteins were separated using SDS-polyacrylamide gel electrophoresis (PAGE) and electro-transferred onto polyvinylidene difluoride (PVDF) membranes (Bio-Rad Laboratories, Richmond, CA). Membranes were blocked with 5% (w/v) low-fat milk for 1 h at room temperature and probed with the primary anti-ETS1, ELK1, ETV4, and β-Tubulin antibodies at 4°C overnight. After washing, membranes were incubated for 1 h at room temperature with horseradish-peroxidase-conjugated secondary antibodies (Jackson ImmunoResearch, Newmarket, United Kingdom). Proteins were detected using Pierce^TM^ enhanced chemiluminescence reagents (Thermo Fisher Scientific).

### Identification of Putative TF Regulators of the Ang-1 Transcriptome

**FIGURE 1:**
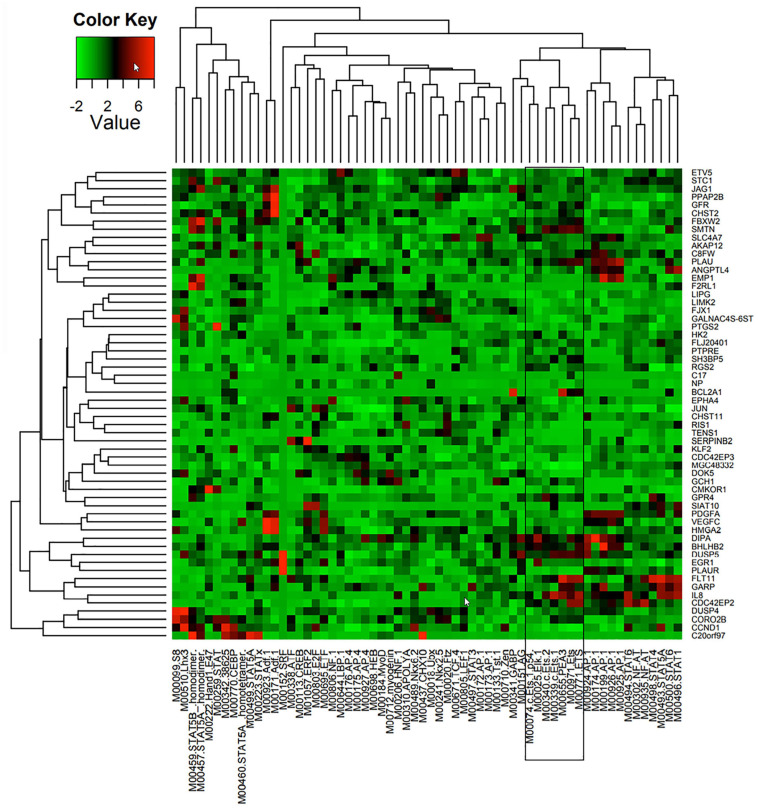
Predicted TF regulators of Ang-1-induced genes in ECs. Heat map representing 58 genes (*Y* axis) that were upregulated in HUVECs in response to 4 h of Ang-1 exposure. Genes are clustered according to Z-scores obtained from 62 position weight matrices generated using TRANSFAC and JASPAR databases and based on flanking regions 10 kb upstream or downstream of each gene (*X* axis). Color scale represents value of Z-score.

A combination of comparative genomics (phylogenetic analysis with space/time models (PHAST) (Siepel et al., 2005) and statistical models (genome-wide analysis of TFBS over-representation (GATOR) (Blanchette et al., 2006) were used to identify likely common regulators of 86 Ang-1-upregulated genes, together with the locations of their putative binding sites. TRANSFAC (Matys et al., 2006) is a commercial database that contains a redundant set of 892 matrices; JASPAR (Portales-Casamar et al., 2010) is an open database that contains a set of 436 matrices. A position weight matrix (PWM) was created using these databases. Transcription factor binding sites (TFBS) that are found in conserved non-coding sequences (CNS), taking into consideration genome-wide binding site frequency, distribution, and GC content biases, are marked as “putatively regulatory” of the gene set. Genes are then clustered based on similar TFBS content, resulting in the prediction of a small number of TFs specifically associated with each gene (Figure 1). Such approaches have been shown to be highly effective at narrowing down a list of candidate TFs pertinent to the process under investigation (Bulyk, 2003; Defrance and Touzet, 2006). In this study, we are interested in a set of ETS TFs that are activated by the Ang-1/Tie-2 pathway. We hypothesize that they significantly contribute to the regulation of the pro-angiogenic processes that are elicited by this pathway, including proliferation, migration, and differentiation.

### Electromobility Shift Assays (EMSA)

Assays were performed using Gel-Shift kits (Panomics, Fremont, CA) with double-stranded DNA probes for human ETS1 and ETV4 and 20 μg of nuclear extract, according to the manufacturer’s instructions.

### TF Reporter Assays (TFRA)

ETS1 and ELK1 reporters were obtained from Dr. M. Jiwaji (University of Glasgow, United Kingdom). DNA encoding a multiple cloning cassette and thymidine kinase promoter (PTK) was inserted between *Kpn*I and *Hin*dIII upstream of the firefly luciferase gene (Fluc) in pGL3-Basic vector (Promega), generating pMN2. DNA encoding consensus TFBS sequences were inserted upstream of PTK. Fluc was then replaced with a unique DNA reporter sequence (UR) so that each TFBS was attached to a different UR (Jiwaji et al., 2012). Briefly, HUVECs were pelleted and re-suspended in Opti-MEM (100 μl) at a density of 10^7^ cells/ml and mixed with 1 μg of *Renilla* luciferase (Rluc) vector (pRL-SV40^®^, Promega) and 1 μg of each of the TFBS encoding vectors (Supplementary Table 2). Cells were electroporated using an Amaxa^®^ nucleofection program. Nucleofected cells were incubated at 37°C for 24 h prior to Ang-1 treatment. HUVECs were then incubated in basic medium containing aliquots of PBS or Ang-1 (300 ng/ml) for 4 h, lysed, and mRNA was extracted as described above. UR and Rluc analyses were conducted as described above. A 10-fold dilution series of UR or Rluc linear dsDNA was created and a standard curve was generated. Unknown samples were compared to the standard curve and the copy number was calculated. Transfection efficiency was accounted for by normalizing UR copy numbers to that of Rluc in each sample. Changes in gene expression were quantified by comparing the log2 ratio for Ang-1 treated cells to PBS treated cells.

### Immunofluorescence

HUVECs were grown on NUNC^TM^ LabTek chamber slides coated with fibronectin. At confluence, cells were maintained in basic medium (2% FBS) for 6 h then exposed to basic medium containing aliquots of PBS or Ang-1 (300 ng/ml) for 1, 3, or 6 h. Cells were fixed in 4% paraformaldehyde for 10 min, permeabilized in 0.5% Triton X-100 in PBS for 10 min, then incubated overnight at 4°C with primary antibodies against ETS1 (1:100), ELK1 (1:800), or ETV4 (1:800). Cells were washed and treated with rhodamine-conjugated goat anti-rabbit secondary antibody (Molecular Probes, Eugene, OR) for 1 h at room temperature then incubated with 4’,6’-diamidino-2-phenylindole (DAPI) for 5 min. Five chambers were used for each condition and six images per chambers were obtained using a confocal microscope (Carl Zeiss Canada, Toronto, ON).

### Transfection With siRNA Oligos

HUVECs were transfected with 10 nM of siGENOME^®^ SMARTpool^®^ synthetic siRNA oligos (Dharmacon, Lafayette, CO) using Lipofectamine RNAiMAX^®^ reagent (Life Technologies). Oligos were selectively directed against ETS1, ELK1, ETV4, or a scrambled siRNA pool. All experiments were performed 48 h post-transfection. The degree of knockdown and selectivity of siRNAs were verified with qPCR.

### Cell Counting Assays

Cell survival was measured by seeding siRNA-transfected HUVECs onto 12-well plates at a density of 8 × 10^4^ cells/cm^2^. Equal numbers of cells were maintained for 24 h in complete (20% FBS) or basic medium (2% FBS) containing aliquots of PBS or Ang-1 (300 ng/ml). Cells were counted using a hemocytometer.

### Cell Migration Assays

EC migration was measured using a scratch (wound) healing assay as previously described (Abdel-Malak et al., 2008b). siRNA-transfected HUVECs were grown as monolayers then wounded with a 200 μl pipette tip. Cells were maintained in basic medium containing PBS or Ang-1 (300 ng/ml) for 8 h. Wounded areas were imaged using an Olympus inverted microscope and quantified using Image-Pro Plus^TM^ software (Media Cybernetics, Bethesda, MD). Values are reported as% wound healing, calculated according to the following formula:

% wound healing = [1—(wound area at t8/wound area at t_0_)] × 100

where t_8_ is the time (8 h) over which cells were maintained in media and t_0_ is the time immediately following wounding.

### Capillary-Like Tube Formation

siRNA-transfected HUVECs were seeded onto 96-well plates pre-coated with growth factor-reduced Matrigel^®^ at a density of 1 × 10^4^ cells per well. Cells were maintained in basic medium containing PBS or Ang-1 (300 ng/ml) for 24 h. Whole-well images were captured using an Olympus inverted microscope (40X) and analyzed using Image-Pro Plus^TM^ software. Angiogenic tube formation was determined by counting the number of tubes formed per field, as previously described (Echavarria and Hussain, 2013).

### Data Analysis

Data are expressed as means ± SEM. Differences between experimental groups were determined using a two-way analysis of variance (ANOVA) followed by a Student–Newman–Keuls *post hoc* test. *P*-values < 0.05 were considered statistically significant.

## Results

### ETS1, ELK1, and ETV4 Are Activated by Ang-1

TFBS enrichment analyses for ETS1, ELK1, and ETV4 generated high Z-scores for genes that are upregulated by Ang-1 (Supplementary Table 3). To identify them as TFs that are likely involved in Ang-1-induced gene regulation, gene-by-gene scoring analysis was performed, resulting in a two-dimensional PWM of Z-scores with 62 columns and 58 rows for the Ang-1-upregulated gene set. It was plotted using the heatmap.2 function from the gplots package of R. A cluster in a heat map reflects how groups of genes are regulated by a TF or a family of TFs. We observed several clusters corresponding to ETS1, ELK1, and ETV4 (Figure 1, Supplementary Figure 1, and Supplementary Spreadsheet 1).

**FIGURE 2:**
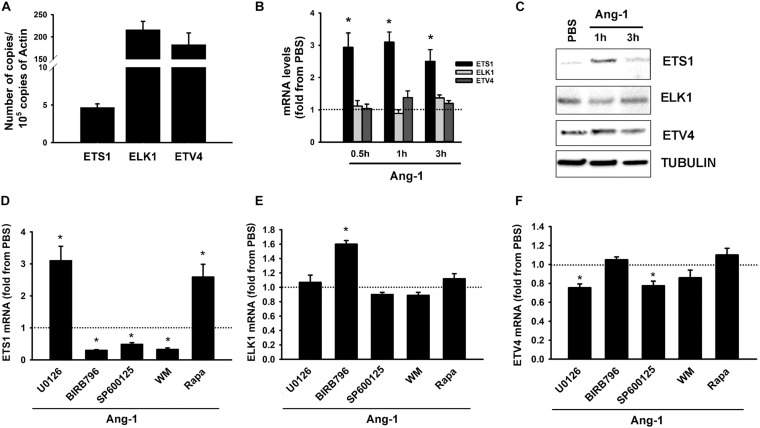
Ang-1 regulates ETS TF expression and DNA binding activity. **(A)** Relative abundance of ETS1, ELK1, and ETV4 mRNA in HUVECs maintained in complete medium. Values are means ± SEM, expressed as number of copies per 10^5^ copies of β-ACTIN. *N* = 4 per group. **(B)** mRNA levels of ETS1, ELK1, and ETV4 in HUVECs exposed to PBS or Ang-1 (300 ng/ml) for 30 min, 1, or 3 h. Values are means ± SEM, expressed as fold relative to PBS. *P < 0.05, compared to PBS. *N* = 6 per condition. **(C)** Protein expression of ETS1, ELK1, and ETV4 in HUVECs exposed to PBS or Ang-1 for 1 h and 3 h. **(D–F)** mRNA levels of ETS1, ELK1, and ETV4 in HUVECs pre-incubated for 1 h with various pathway inhibitors then exposed to PBS or Ang-1 for 1 h. Values are means ± SEM, expressed as fold from PBS. *P < 0.05, compared to PBS. *N* = 6 per condition.

Basal mRNA measurements indicate that ELK1 and ETV4 are relatively more abundant in ECs than ETS1 is (Figure 2A). Ang-1 upregulated ETS-1 at 30 min, 1, and 3 h relative to PBS, but had no effect on ELK1 or ETV4 mRNA levels at any time point (Figure 2B). Ang-1 upregulated ETS1 protein expression at 1 h relative to PBS but had no effects on ELK1 or ETV4 protein levels (Figure 2C). Effects of various pathway inhibitors on basal ETS1, ELK1, and ETV4 mRNA levels are shown in Supplementary Figure 2. When p38 and SAPK/JNK pathways were inhibited, basal ETS1 mRNA levels mildly but significantly decreased. Basal ELK1 and ETV4 mRNA levels were not altered by various pathway inhibitors (Supplementary Figure 2). When the ERK1/2 and mTOR pathways were inhibited, Ang-1 upregulated ETS1, relative to PBS. When the p38, SAPK/JNK, and PI-3 kinase pathways were inhibited, Ang-1 downregulated ETS1 (Figure 2D). When the ERK1/2, SAPK/JNK, PI-3 kinase, and mTOR pathways were inhibited, Ang-1 had no effect on ELK1 but when the p38 pathway was inhibited, Ang-1 upregulated ELK1 (Figure 2E). When the p38, PI-3 kinase, and mTOR pathways were inhibited, Ang-1 had no effect on ETV4 but when the ERK1/2 and SAPK/JNK pathways were inhibited, Ang-1 downregulated ETV4 (Figure 2F). These results demonstrate that, in ECs, the p38, SAPL/JNK, and PI-3 kinase pathways are involved in Ang-1-induced transcriptional activation of ETS1, the p38 pathway exerts an inhibitory effect on ELK1 expression, and the ERK1/2 and SAPK/JNK pathways stimulate ETV4 expression.

**FIGURE 3:**
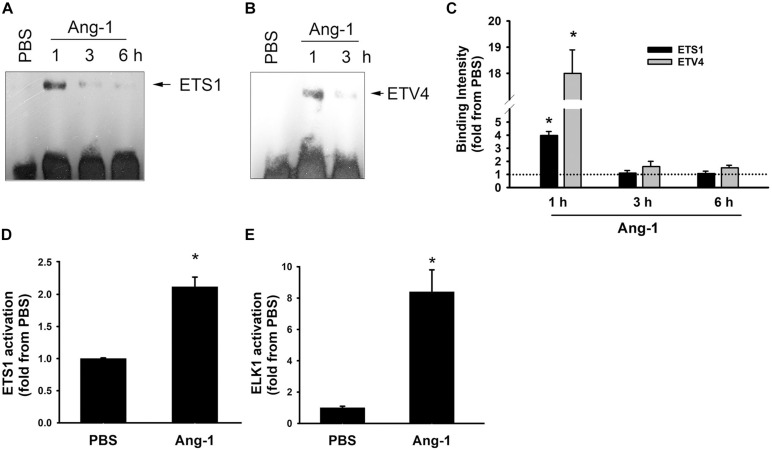
Ang-1 activates ETS1, ELK1, and ETV4 TFs. **(A–C)** Representative examples of electrophoretic mobility shift assays and mean values of probe binding intensity (expressed as fold from PBS) of ETS1 and ETV4 in response to PBS or Ang-1 exposure. *N* = 3. **(D,E)** Luciferase reporter activities of ETS1 and ELK1 in HUVECs exposed to PBS or Ang-1 for 4 h. Values are means ± SEM, expressed as fold from PBS. *P < 0.05, compared to PBS. *N* = 6 per condition.

Electrophoretic mobility shift revealed that DNA binding activities of ETS1 and ETV4 increased by 4- and 18-fold, respectively, 1 h post Ang-1 exposure with a decline thereafter to levels that are similar to those of PBS (Figures 3A–C). Luciferase reporter assays showed that DNA binding activities of ETS1 and ELK1 increased in response to Ang-1 exposure (Figures 3D,E).

### Intracellular Mobilization

**FIGURE 4:**
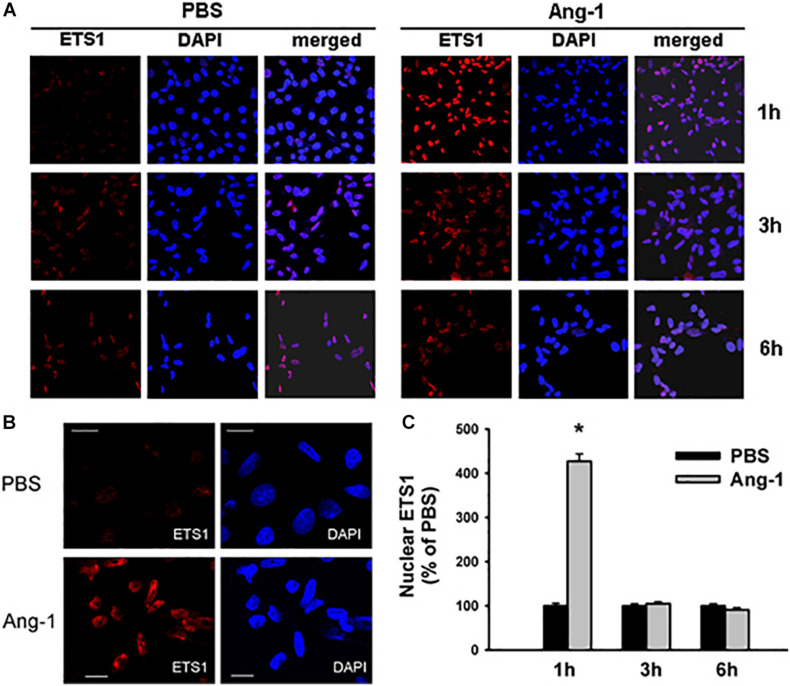
Ang-1 increases nuclear localization of ETS1. **(A)** Representative immunofluorescence images of ETS1 protein (red color) and nuclear staining (DAPI, blue) in HUVECs exposed to PBS or Ang-1 for 1, 3, or 6 h. **(B)** ETS1 and DAPI staining in HUVECs exposed to PBS or Ang-1 for 1 h. **(C)** Fluorescence intensity of nuclear ETS1 in HUVECs exposed to PBS or Ang-1 for 1, 3, and 6 h. Values are means ± SEM, expressed as percent of PBS. *P < 0.05, compared to PBS. *N* > 130 cells per group. White bars in panel B indicate 20 μm.

**FIGURE 5:**
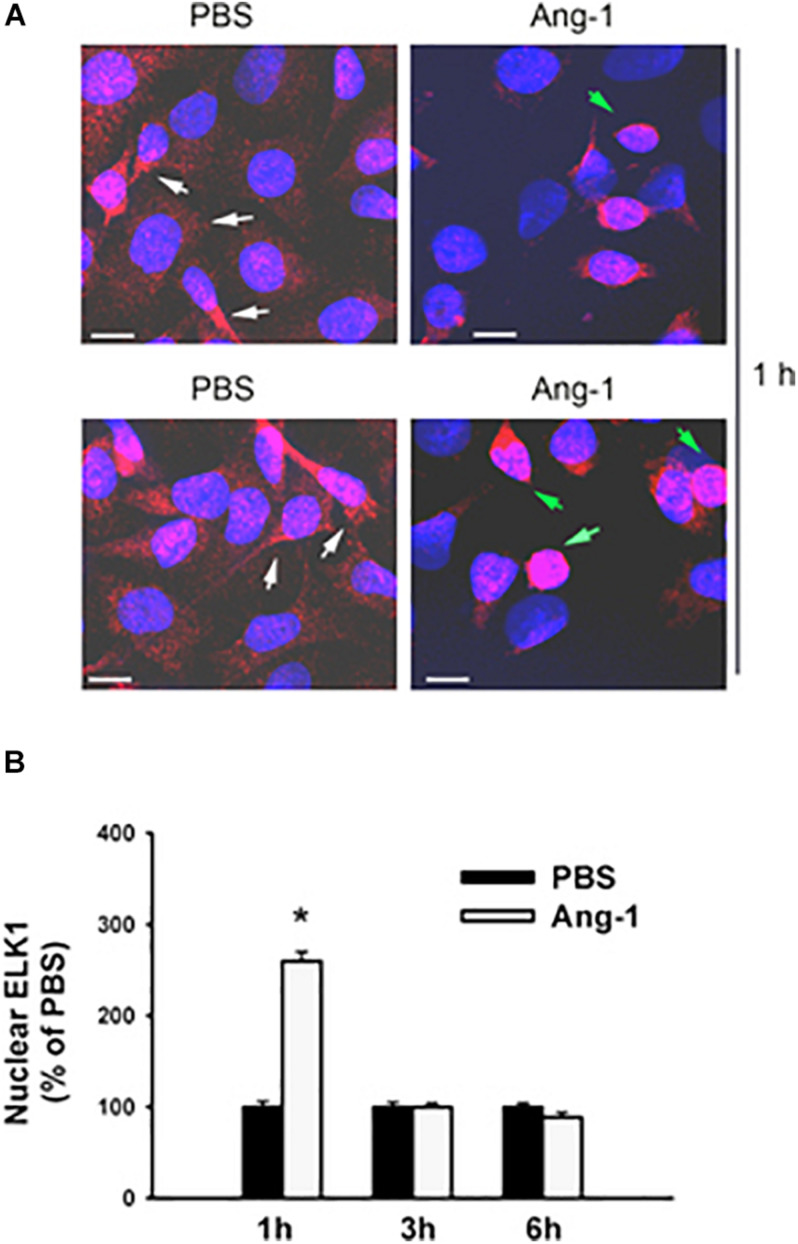
Ang-1 increases nuclear localization of ELK1. **(A)** Representative immunofluorescence images of ELK1 protein (red color) and nuclear staining (DAPI, blue) in HUVECs exposed to PBS or Ang-1 for 1 h. Green arrows indicate cells with strong nuclear ELK1 staining; white arrows indicate cells with strong cytosolic ELK1 staining. **(B)** Fluorescence intensity of nuclear ELK1in HUVECs exposed to PBS or Ang-1 for 1, 3, or 6 h. Values are means ± SEM, expressed as percent of PBS. *P < 0.05, compared to PBS. *N* > 130 cells per group. White bars indicate 10 μm.

**FIGURE 6:**
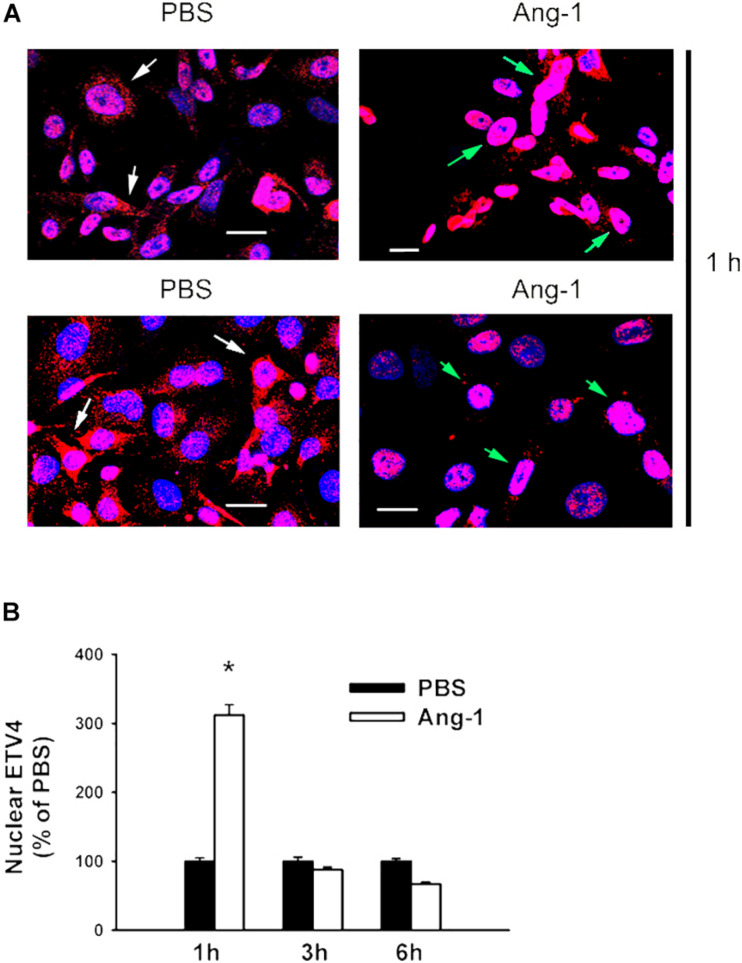
Ang-1 increases nuclear localization of ETV4. **(A)** Representative immunofluorescence images of ETV41 protein (red color) and nuclear staining (DAPI, blue) in HUVECs exposed to PBS or Ang-1 for 1 h. Green arrows indicate cells with nuclear ETV4 staining; white arrows indicate cells with strong cytosolic ETV4 staining. **(B)** Fluorescence intensity of nuclear ETV4 in HUVECs exposed to PBS or Ang-1 for 1, 3, or 6 h. Values are means ± SEM, expressed as percent of PBS. *P < 0.05, compared to PBS. *N* > 130 cells per group. White bars indicate 20 μm.

Immunofluorescence microscopy showed that in cells exposed to PBS, ETS1 protein was strictly expressed in the nucleus (Figure 4). Exposure to Ang-1 elicited a fourfold increase in the intensity of nuclear ETS1 protein relative to PBS (Figure 4). This induction was evident 1 h post Ang-1 exposure with a decline to control levels (Figure 4). In cells exposed to PBS, ELK1 and ETV4 proteins were detected in the cytoplasm (white arrows) and the nucleus (green arrows) (Figures 5, 6). Nuclear intensities of ELK1 and ETV4 increased by 2.5- and 3-fold, respectively, 1 h post-Ang-1 with a decline thereafter to control levels (Figures 5, 6).

### Regulation of EC Survival, Migration, and Differentiation

**FIGURE 7:**
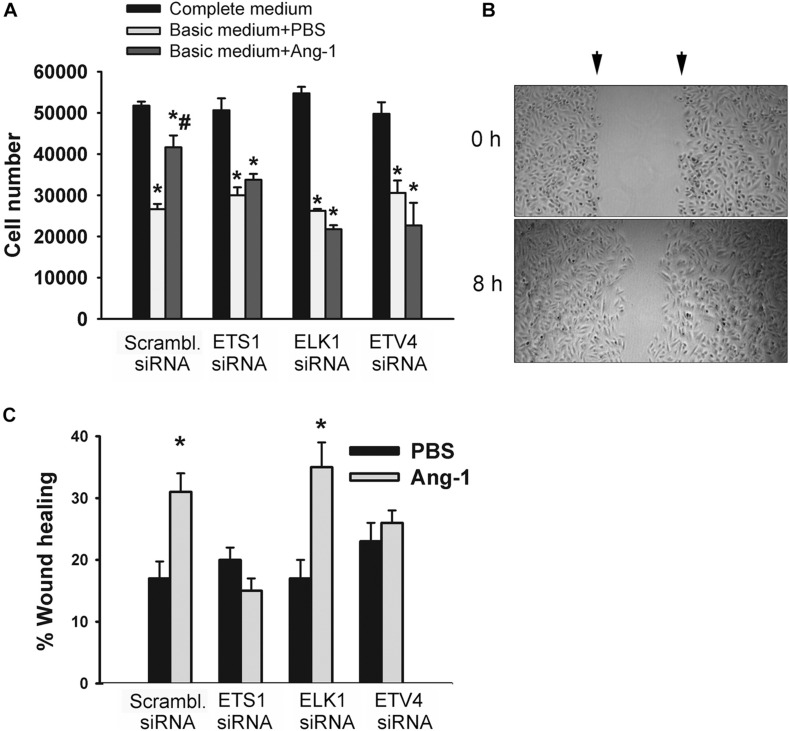
ETS1, ELK1, and ETV4 contribute to Ang-1-induced EC survival and migration. **(A)** Cell counts of HUVECs transfected with scrambled, ETS1, ELK1, or ETV4 siRNA oligos. Equal numbers of cells were maintained in complete (20% FBS), basic medium (2% FBS) containing PBS, or Ang-1 (300 ng/ml). Cells counted 24 h later. Values are means ± SEM. *P < 0.05, compared to complete medium. ^#^*P* < 0.05, compared to PBS. **(B)** Representative examples of scratch wound healing assays in HUVECs transfected with scrambled siRNA and maintained in basic medium containing Ang-1. Arrows indicate wound margins. **(C)** Scratch wound healing in HUVECs transfected with scrambled, ETS1, ELK1, or ETV4 siRNA oligos and maintained in basic medium containing PBS or Ang-1. Percent wound healing measured 8 h after wounding with pipette tip. Values are means ± SEM. *P < 0.05, compared to PBS.

The degree of ETS1, ELK1, and ETV4 knockdown and selectivity of siRNAs are shown in Supplementary Figure 3. Cell counts of scrambled siRNA-transfected ECs decreased when incubated in basic medium containing PBS, relative to complete medium (Figure 7A and Supplementary Figure 4). Counts were higher when cells were incubated in basic medium containing Ang-1, relative to PBS (Figure 7A and Supplementary Figure 4). These results suggest that Ang-1 exerts a pro-survival effect on cells. When cells were transfected with ETS1, ELK1, and ETV4 siRNAs, counts did not increase in response to Ang-1, suggesting that they are required for Ang-1-induced EC survival (Figure 7A and Supplementary Figure 4).

Migration of scrambled siRNA-transfected ECs increased in response to Ang-1, relative to PBS. This suggests that Ang-1 exerts a pro-migration effect on cells. When cells were transfected with ETS1, and ETV4 siRNAs, migration did not increase in response to Ang-1. When cells were transfected with ELK1 siRNA, migration increased in response to Ang-1. These results suggest that ETS1 and ETV4 contribute to Ang-1-induced EC migration (Figure 7B).

**FIGURE 8:**
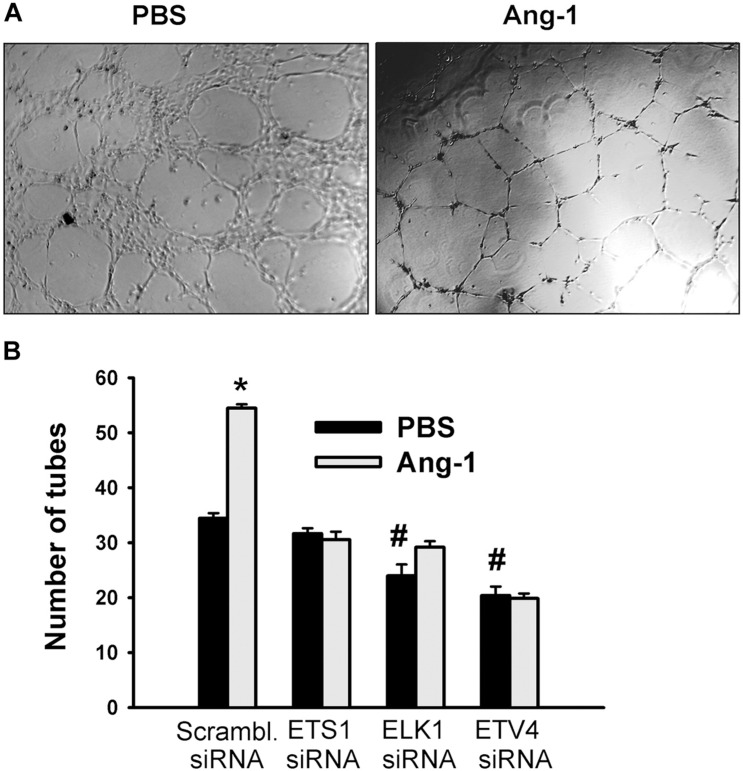
ETS1, ELK1, and ETV4 contribute to Ang-1-induced EC differentiation. **(A)** Representative images of capillary-like tube formation of HUVECs transfected with siRNA oligos and maintained for 24 h on plates pre-coated with growth factor-reduced Matrigel and basic medium containing PBS or Ang-1. **(B)** Total tube number of HUVECs transfected with siRNA oligos and maintained for 24 h on plates pre-coated with growth factor-reduced Matrigel and basic medium containing PBS or Ang-1. Values are means ± SEM. *N* = 8 per group. *P < 0.05, compared to PBS. ^#^*P* < 0.05, compared to scrambled siRNA oligo-transfected cells treated with PBS.

Differentiation into capillary-like tube structures of scrambled siRNA-transfected ECs increased in response to Ang-1, relative to PBS. This suggests that Ang-1 exerts a pro-differentiation effect on cells. When cells were transfected with ETS1, ELK1, and ETV4 siRNAs, differentiation did not increase in response to Ang-1, relative to PBS. With ELK1 and ETV4, basal (PBS) differentiation decreased when compared to scrambled siRNA levels. These results suggest that ETS1, ELK1, and ETV4 are required for Ang-1-induced EC differentiation and that ELK1 and ETV4 regulate basal differentiation (Figure 8).

### ETS1, ELK1, and ETV4 Regulation of Angiogenesis-Related Gene Expression

**FIGURE 9:**
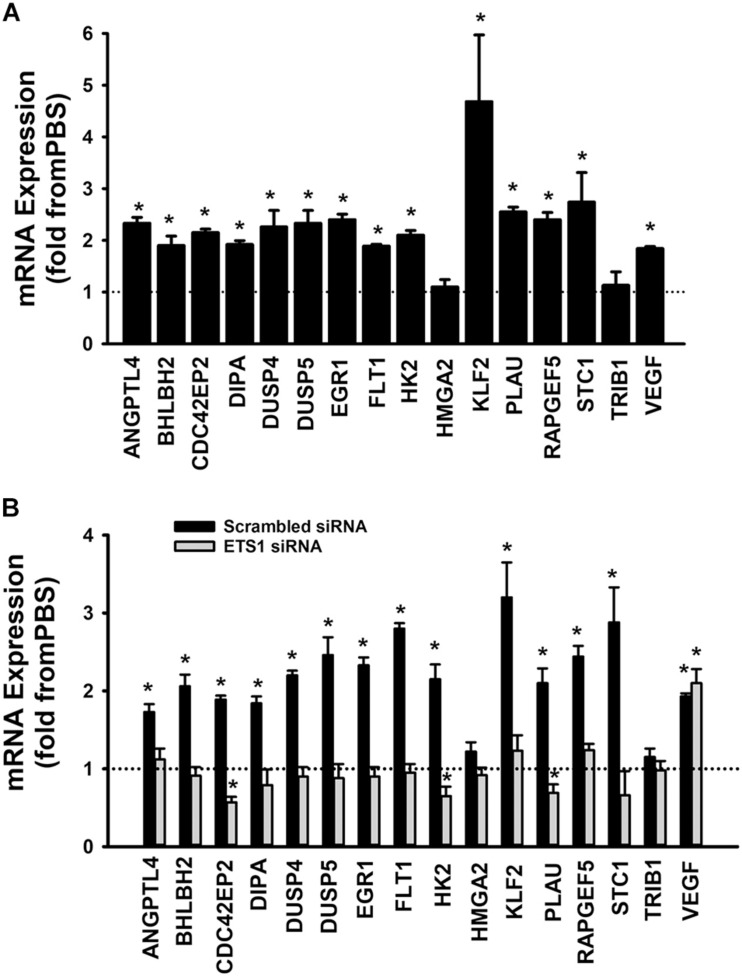
Contribution of ETS1 to Ang-1-induced gene expression. **(A)** Effects of Ang-1 (300 ng/ml) on mRNA expressions of various genes in HUVECs. Cells maintained in basic medium containing PBS or Ang-1 for 4 h. Values are means ± SEM, expressed as fold from PBS. *P < 0.05, compared to PBS. *N* = 8 per group. **(B)** Effects of Ang-1 (300 ng/ml) on mRNA expressions of various genes in HUVECs transfected with scrambled or ETS1 siRNA oligos. Cells maintained in basic medium containing PBS or Ang-1 for 4 h. Values are means ± SEM, expressed as fold from PBS. *P < 0.05, compared to PBS. *N* = 8 per group.

A set of fifteen genes (ANGPTL4, BHLBH2, CDC42EP2, DIPA, DUSP4, DUSP5, EGR1, FLT1, HK2, HMGA2, KLF2, PLAU, RAPGEF5, STC1, and TRIB1) was selected from the Ang-1 transcriptome according to the following criteria: (a) they are upregulated by Ang-1; (b) they were predicted to be transcriptionally regulated by ETS TFs; and (c) they possess angiogenesis-related biological functions as annotated by the Gene Ontology (GO) bioinformatics network (Wang et al., 2011). In response to 4 h of exposure to Ang-1, the expressions of all thirteen genes and VEGF-A were upregulated (Figure 9A). Expression of HMGA2 and TRIB1 were not altered by Ang-1 suggesting that the previously reported upregulation of their expression in response to Ang-1 was a false positive finding (Abdel-Malak et al., 2007). VEGF was included because of its importance in angiogenesis. When scrambled siRNA-transfected ECs were exposed to 4 h of exposure Ang-1, the expressions of all thirteen genes and VEGF-A were still upregulated (Figure 9B).

**FIGURE 10:**
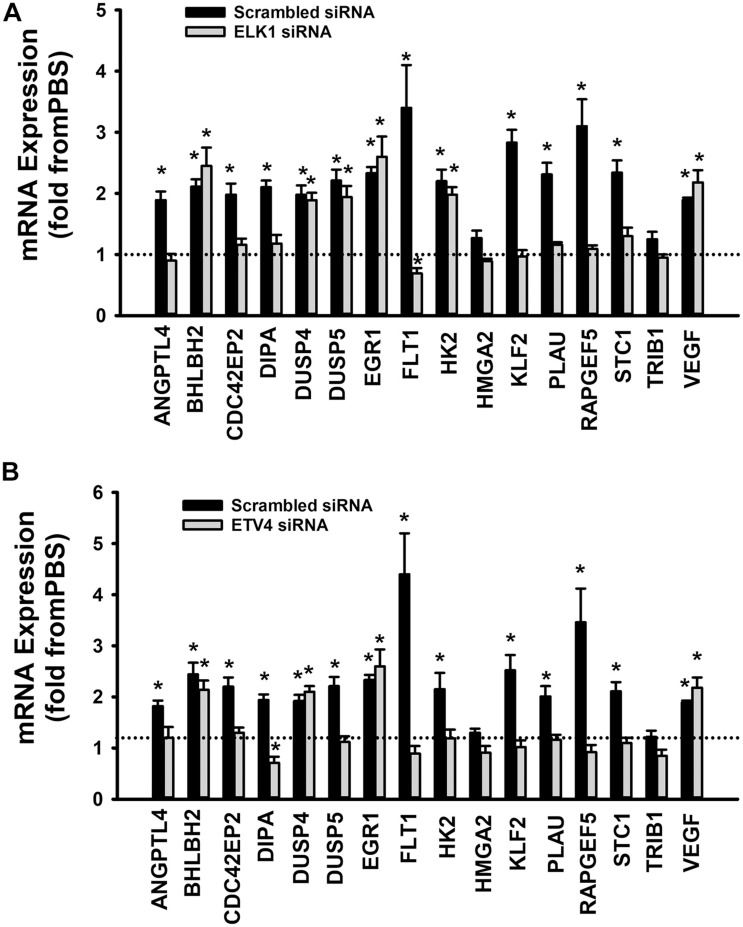
Contributions of ELK1 and ETV4 to Ang-1-induced gene expression. **(A)** Effects of Ang-1 (300 ng/ml) on mRNA expressions of various genes in HUVECs transfected with scrambled or ELK1 siRNA oligos. Cells maintained in basic medium containing PBS or Ang-1 for 4 h. Values are means ± SEM, expressed as fold from PBS. *P < 0.05, compared to PBS. *N* = 6 per group. **(B)** Effects of Ang-1 (300 ng/ml) on mRNA expressions of various genes in HUVECs transfected with scrambled or ETV4 siRNA oligos. Cells maintained in basic medium containing PBS or Ang-1 for 4 h. Values are means ± SEM, expressed as fold from PBS. *P < 0.05, compared to PBS. *N* = 6 per group.

When cells were transfected with ETS1 siRNA, Ang-1 upregulated VEGF expression but did not exert any regulatory effects on the other thirteen genes (Figure 9B). Expression of HMGA2 and TRIB1 were not altered by transfection with ETS1 siRNA (Figure 9B). CDC42EP2, HK2, and PLAU expressions were lower in response to Ang-1 exposure than they were with PBS (Figure 9B). When cells were transfected with ELK1 siRNA, Ang-1 did not exert any regulatory effects on ANGPTL4, CDC42EP2, DIPA, FLT1, HMGA2, KLF2, PLAU, RAPGEF2, STC1, or TRIB1 but upregulated BHLBH2, DUSP4, DUSP5, EGR1, HK2, and VEGF expressions (Figure 10A). FLT1 expression was lower in response to Ang-1 exposure than it was with PBS (Figure 10A). When cells were transfected with ETV4 siRNA, Ang-1 did not exert any regulatory effects on ANGPTL4, CDC42EP2, DIPA, FLT1, HK2, HMGA2, KLF2, PLAU, RAPGEF2, STC1, or TRIB1, but upregulated BHLBH2, DUSP4, EGR1, and VEGF expressions (Figure 10B). DIPA expression was lower in response to Ang-1 exposure than it was with PBS (Figure 10B). Since ETS1, ELK1, and ETV4 knockdown inhibited Ang-1-induced upregulation of thirteen, eight, and nine pro-angiogenesis genes, respectively, we conclude that they play significant angiogenic roles in Ang-1 signaling in ECs.

## Discussion

In this study, we characterized the regulatory roles of three members of the ETS family of TFs in Ang-1 signaling and the angiogenic response in ECs. Our study demonstrates for the first time that: (1) Ang-1 induces ETS1 mRNA and protein expression, increases nuclear translocation of ETS1, ELK1, and ETV4, and activates their DNA binding activities; (2) ETS1, ELK1, and ETV4 play important roles in Ang-1-induced EC cell survival and differentiation; ETS1 and ETV4 are required for Ang-1-induced EC migration; and (3) ETS1, ELK1, and ETV4 regulate the expressions of several angiogenesis-related genes downstream from the Ang-1/Tie-2 receptor pathway.

### ETS1, ELK1, and ETV4 Expression and Activity

There is vast literature regarding the expression and possible roles of ETS transcription factors in hematological malignancies and solid tumors (Sizemore et al., 2017). Elevated expression of several members of ETS family including ERG, ETV1, ETV4, and ETV5 is considered as a common event in human prostate cancer and this overexpression has been attributed to chromosomal rearrangements involving the fusion of the androgen-activated gene TMPRSS2 with ETS transcription factors (Clark and Cooper, 2009). ETS gene rearrangements have also been proposed as a key event causing prostate neoplastic development (Mesquita et al., 2015; Sizemore et al., 2017).

Little information is available regarding the action of transcription factors that mediate the biological activity of the Ang-1/Tie-2 receptor pathway in ECs. Previous reports had shown that ETS transcription factors are activated by several pro-angiogenesis growth factors (Randi et al., 2009) and that Ang-1 induces ETS-1 expression in peripheral blood stem cells (Kim et al., 2009), but their roles in Ang-1/Tie-2 receptor signaling and the angiogenic response in ECs had not been explored. To our knowledge, our group was the first to report that Ang-1 increases ELK1 phosphorylation in HUVECs (Harfouche et al., 2003). However, neither DNA binding capacity, the nuclear mobilization of ELK1, or the functional importance of ELK1 in Ang-1-induced angiogenic processes were evaluated in that study. We report here that Ang-1 upregulates ETS1 expression and enhances DNA binding activities of ETS1, ELK1, and ETV4 in ECs.

The mitogen-activated protein kinase (MAPK) and PI-3 kinase/AKT pathways play important roles in Ang-1-induced EC survival, proliferation, migration, and adhesion (Kim et al., 2000; Harfouche et al., 2002; Harfouche et al., 2003; Brindle et al., 2006). In ECs, members of the MAPK family of enzymes are important activators of ETS TFs. For instance, VEGF upregulates ETS1 through the ERK1/2 and protein kinase C pathways in retinal ECs (Watanabe et al., 2004). ELK1 is activated by the ERK1/2, p38, and SAPK/JNK pathways, with each pathway targeting a different ELK1 domain (Yang et al., 1998). The p38 and SAPK/JNK pathways activate ETV4 (Selvaraj et al., 2015). We report that Ang-1-induced upregulation of ETS1 in HUVECs is dependent on the p38, SAPK/JNK, and PI-3 kinase/AKT pathways. When these pathways were inhibited, ETS1 expression decreased in response to Ang-1 exposure (Figure 2D). Analyses of the promoter of human ETS1 indicate the presence of binding sites for AP-1, AP-2, SP-1, and ETS1 transcription factors and that c-Jun, rather than combination of c-Jun and c-Fos, activates the promoter (Oka et al., 1991). We have previously reported that c-Jun protein is activated downstream from Tie-2 receptors in ECs (Abdel-Malak et al., 2008b). Based on these observations, we speculate that the dependence of basal levels of ETS1 and Ang-1-induced ETS1 expression on the p38 and SAPK/JNK pathways is the result of activation of c-Jun by these two pathways and the subsequent binding of c-Jun to the ETS1 promoter. The role of the PI-3 kinase pathway in ETS1 regulation is not well documented in ECs. In vascular smooth muscles, PDGF-BB induces ETS1 expression through the PI-3 kinase/AKT and mTOR pathways (Lo et al., 2009). Our study shows that the PI-3 pathway is required for ETS1 induction downstream from Tie-2 receptors but not for basal ETS1 expression.

Although ELK1 expression was not affected by Ang-1 when the EKR1/2, SAPK/JNK, PI-3 kinase, and mTOR pathways were inhibited, Ang-1 upregulated ELK1 when the p38 pathway was inhibited (Figure 2E). We speculate that this response was mediated by enhanced ERK1/2 activity, which was compensatory for p38 inhibition. Indeed, we have previously noted that the p38 pathway inhibits ERK1/2 phosphorylation and activation and that when this inhibition is removed by a selective p38 inhibitor, ERK1 and ERK2 proteins are strongly activated and upregulate several important angiogenesis-related genes in ECs, including the transcription factor EGR1. Westermarck et al. (2001) attributed p38 inhibition of ERK1/2 to the activation of protein phosphatases 1 (PP1) and 2A (PP2A), which selectively target upstream regulators of ERK1 and ERK2 proteins.

We observed that ETV4 expression was downregulated by Ang-1 when the ERK1/2 and SAPK/JNK pathways were inhibited (Figure 2F). Little information is available regarding MAPK regulation of ETV4 expression in ECs. In neurons, nerve growth factor (NGF) stimulates ETV4 expression through the ERK1/2 pathway (Fontanet et al., 2013), while it is regulated by both the ERK1/2 and SAPK/JNK pathways in cancer cells (O’Hagan et al., 1996). Guo and Sharrocks (2009) reported that the ERK1/2 pathway promotes sumoylation of ETV4 protein and that this post-translational modification is required for maximal activation of ETV4 DNA binding activity. In the current study, we report that both the ERK1/2 and SAPK/JNK pathways are important for maintaining ETV4 expression in ECs and that their inhibition is associated with significant downregulation of ETV4. There is evidence that ERK1/2 and SAPK/JNK pathways are strongly activated in a variety of human tumors, including glioblastoma, kidney, colon, and breast tumors and that both pathways contribute to tumor angiogenesis (Sivaraman et al., 1997; Hoshino et al., 1999; Ennis et al., 2005; Huang et al., 2008). It has also been shown that MAPKs are involved in retinal angiogenesis (Bullard et al., 2003). Our current study suggests that the roles of ERK1/2 and SAPK/JNK pathways in pathological angiogenesis may be mediated through upregulation and activation of ETV4 in ECs and that this TF should be considered as a possible therapeutic target.

### Regulation of EC Survival, Migration, and Differentiation by ETS TFs

It has been well established that members of the ET family of TFs regulate angiogenesis. Indeed, it has been reported that ETS1, ELK1, and ETV4 positively regulate angiogenic processes such as migration and differentiation downstream from several growth factors (Iwasaka et al., 1996; Sato, 2001; Sato et al., 2001; Randi et al., 2009). It should be noted, however, that the role of ETS1 in EC survival and apoptosis remain debatable since both pro- and anti-apoptotic roles have been reported. Its pro-apoptotic effect was attributed to upregulation of several pro-apoptosis genes, including BID, p21, p27, and caspase-4 (Teruyama et al., 2001). Our research demonstrates for the first time that ETS1 mediates Ang-1-induced EC migration and differentiation and that ETV4 plays an important role in Ang-1-induced EC migration. Although we did not measure apoptosis in this study, the fact that the pro-survival effect of Ang-1 was absent when ETS TF expressions were knocked down suggests that they contribute to Ang-1-induced EC survival (Figure 7). Finally, our results indicate that Ang-1-induced EC differentiation into tubes also requires the presence of ELK1 and ETV4 (Figure 8).

To identify the mechanisms through which ETS1, ELK1, and ETV4 regulate Ang-1-induced angiogenesis, we measured how their selective downregulation affected the expressions of a set of thirteen angiogenesis-related genes selected from a published list of the Ang-1-regulated transcriptome. In ECs transfected with scrambled siRNA, Ang-1 upregulated the expressions of ANGPTL4, BHLBH2, CDC42EP2, DIPA, DUSP4, DUSP5, EGR1, FLT1, HK2, KLF2, PLAU, RAPGEF5, and STC1, relative to PBS (Figure 9A). Ang-1 did not upregulate these genes in ETS1 knockdown cells, suggesting that ETS1 is required for Ang-1-induced transcriptional regulation of these genes (Figure 9B). Similarly, ELK1 is required for Ang-1-induced upregulation of eight of these genes, including ANGPTL4, CDC42EP2, DIPA, FLT1, KLF2, PLAU, RRAPGEF5, and STC1 (Figure 10A), and ETV4 is required for Ang-1-induced upregulation of nine of these genes, including ANGPTL4, CDC42EP2, DIPA, FLT1, HK2, KLF2, PLAU, RRAPGEF5, and STC1 (Figure 10B). These results suggest that although ETS1 is less abundant than ELK1 or ETV4 in HUVECs, its role in the regulation of angiogenesis-related genes of the Ang-1 transcriptome is relatively stronger than that of the other two. Nevertheless, despite regulating more genes, the contributions of ETS1 to Ang-1-induced survival, migration, and differentiation are qualitatively like those of ELK1 and ETV4. We should emphasize that the gene set that we used contains several well-known positive regulators of EC survival, migration, and differentiation, including ANGPTL4, FLT1, HK2, and PLAU (Shibuya, 2006; Montuori and Ragno, 2014; Mousavizadeh et al., 2016). We have previously identified other genes from the set, such as EGR1, DUSP4, and DUSP5, as being important positive modulators of Ang-1-induced angiogenesis (Abdel-Malak et al., 2008a; Echavarria and Hussain, 2013). Based on the strong pro-angiogenic attributes of many members of the thirteen-gene set, we theorize that ETS1, ELK1, and ETV4 promote Ang-1-induced EC survival, migration, and differentiation through induction of these genes. We should point out that this theory has some limitations. First, our study does not clarify whether ETS1, ELK1, or ETV4 directly or indirectly regulate the above-mentioned genes, although previous reports indicate that PLAU, FLT1, and EGR1 are direct gene targets of ETS1 (Kitange et al., 1999; Valter et al., 1999; Lee et al., 2008). It is possible that all three TFs indirectly regulate angiogenesis-related gene expression by upregulating secondary mediators. Second, it is also possible that ETS, ELK1, and ETV4 contribute to Ang-1-induced EC survival, migration, and differentiation by regulating genes other than those we selected for. Indeed, previous reports indicate that ETS1 promotes angiogenesis through upregulation of integrin β3, and matrix metalloproteinases 1, 3, and 9 (MMP1, MMP3, and MMP9) (Sato et al., 2001). Third, although the majority of ETS TFs bind to DNA as monomers, they directly interact with other TFs, including the Pax family, SP-1, NFκB, STAT5, and AP-1. These interactions determine the transcriptional activities of ETS TFs in regulating distinct genes (Li et al., 2000), so it is possible that ETS1, ELK1, and ETV4 regulation of angiogenesis-related genes downstream from Tie-2 receptors is dependent on unique interactions with TFs other than those belonging to the ETS family at specific binding sites of the promotors of each gene.

### Limitations of the Study

Our study has two major limitations. First, only mRNA levels of ETS1, ELK1, and ETV4 were measured in the pathway inhibitors experiments. Moreover, we only measured mRNA levels of various genes which are regulated by these TFs in Figures 9, 10. Future studies should consider the effects of pathway inhibitor effects on protein levels of ETS1, ELK1, ELK1, and ETV4 as well on post-translational modifications, such as phosphorylation, acetylation, and ubiquitination (Charlot et al., 2010). In addition, protein levels of various genes which are regulated by ETS1, ELK1, and ETV4 should also be evaluated. Second, ETS1, ELK1, and ETV4 activation by the Ang-1/Tie-2 pathway was evaluated using EMSA and luciferase reporter assays that provide qualitative and indirect evidence of ETS TF involvement in the regulation of the genes listed in Figures 9, 10. Further experimentation using chromatin immunoprecipitation (ChIP) assays would demonstrate direct binding of ETS1, ELK1, and ETV4 to the promoters of these genes.

## Conclusion

Our results suggest that ETS1, ELK1, and ETV4, three members of the ETS family of TFs, are activated in ECs in response to Ang-1 exposure and that these TFs may play significant roles in Ang-1-induced EC survival, migration, and differentiation.

## Data Availability Statement

The raw data supporting the conclusions of this article will be made available by the authors, without undue reservation.

## Author Contributions

MB and SHu: conceptualization, supervision. MB, SHa, VS, AM, and DM: methodology. MB, JS-G: software. SHa: validation. SHa, VS, and DM: formal analysis. SHa and VS: data curation. SHa: writing—original draft preparation. SHa, AM, MB, MA, SHa, and JS-G: writing—review and editing. SHa, AM, and SHu: visualization. All authors have read and agreed to the published version of the manuscript.

## Conflict of Interest

The authors declare that the research was conducted in the absence of any commercial or financial relationships that could be construed as a potential conflict of interest.

## Publisher’s Note

All claims expressed in this article are solely those of the authors and do not necessarily represent those of their affiliated organizations, or those of the publisher, the editors and the reviewers. Any product that may be evaluated in this article, or claim that may be made by its manufacturer, is not guaranteed or endorsed by the publisher.

## References

[B1] Abdel-Malak N. A. Mofarrahi M. Mayaki D. Khachigian L. M. Hussain S. N. (2008a). Early Growth Response-1 regulates angiopoietin-1-induced endothelial cell proliferation, migration, and differentiation. *Arterioscler. Thromb. Vasc. Biol.* 29 209–216. 10.1161/atvbaha.108.181073 19112164

[B2] Abdel-Malak N. A. Srikant C. B. Kristof A. S. Magder S. A. Di Battista J. A. Hussain S. N. A. (2008b). Angiopoietin-1 promotes endothelial proliferation and migration through AP-1 dependent autocrine production of interleukin-8. *Blood* 111 4145–4154. 10.1182/blood-2007-08-110338 18252863

[B3] Abdel-Malak N. Harfouche R. Hussain S. N. (2007). Transcriptome of angiopoietin 1-activated human umbilical vein endothelial cells. *Endothelium* 14 285–302. 10.1080/10623320701678268 18080866

[B4] Birdsey G. M. Dryden N. H. Amsellem V. Gebhardt F. Sahnan K. Haskard D. O. (2008). Transcription factor Erg regulates angiogenesis and endothelial apoptosis through VE-cadherin. *Blood* 111 3498–3506. 10.1182/blood-2007-08-105346 18195090 PMC2275018

[B5] Blanchette M. Bataille A. R. Chen X. Poitras C. Laganiere J. Lefebvre C. (2006). Genome-wide computational prediction of transcriptional regulatory modules reveals new insights into human gene expression. *Genome Res.* 16 656–668. 10.1101/gr.4866006 16606704 PMC1457048

[B6] Bolon M. L. Kidder G. M. Simon A. M. Tyml K. (2007). Lipopolysaccharide reduces electrical coupling in microvascular endothelial cells by targeting connexin40 in a tyrosine-, ERK1/2-, PKA-, and PKC-dependent manner. *J. Cell Physiol.* 211 159–166. 10.1002/jcp.20928 17149706

[B7] Brindle N. P. Saharinen P. Alitalo K. (2006). Signaling and functions of angiopoietin-1 in vascular protection. *Circ. Res.* 98 1014–1023. 10.1161/01.res.0000218275.54089.12 16645151 PMC2270395

[B8] Bullard L. E. Qi X. Penn J. S. (2003). Role for extracellular signal-responsive kinase-1 and -2 in retinal angiogenesis. *Invest. Ophthalmol. Vis. Sci.* 44 1722–1731. 10.1167/iovs.01-1193 12657614

[B9] Bulyk M. L. (2003). Computational prediction of transcription-factor binding site locations. *Genome Biol.* 5:201. 10.1186/gb-2003-5-1-201 PMC395725 14709165

[B10] Charlot C. Dubois-Pot H. Serchov T. Tourrette Y. Wasylyk B. (2010). A review of post-translational modifications and subcellular localization of Ets transcription factors: possible connection with cancer and involvement in the hypoxic response. *Methods Mol. Biol.* 647 3–30. 10.1007/978-1-60761-738-9_1 20694658

[B11] Christensen R. A. Fujikawa K. Madore R. Oettgen P. Varticovski L. (2002). NERF2, a member of the Ets family of transcription factors, is increased in response to hypoxia and angiopoietin-1: a potential mechanism for Tie2 regulation during hypoxia. *J. Cell Biochem.* 85 505–515. 10.1002/jcb.10148 11967990

[B12] Clark J. P. Cooper C. S. (2009). ETS gene fusions in prostate cancer. *Nat. Rev. Urol.* 6 429–439. 10.1038/nrurol.2009.127 19657377

[B13] Defrance M. Touzet H. (2006). Predicting transcription factor binding sites using local over-representation and comparative genomics. *BMC Bioinformatics* 7:396. 10.1186/1471-2105-7-396 16945132 PMC1570149

[B14] Echavarria R. Hussain S. N. (2013). Regulation of angiopoietin-1/Tie-2 receptor signaling in endothelial cells by dual-specificity phosphatases 1, 4, and 5. *J. Am. Heart Assoc.* 2:e000571. 10.1161/JAHA.113.000571 PMC3886752 24308939

[B15] Ennis B. W. Fultz K. E. Smith K. A. Westwick J. K. Zhu D. Boluro-Ajayi M. (2005). Inhibition of tumor growth, angiogenesis, and tumor cell proliferation by a small molecule inhibitor of c-Jun N-terminal kinase. *J. Pharmacol. Exp. Ther.* 313 325–332. 10.1124/jpet.104.078873 15626722

[B16] Fontanet P. Irala D. Alsina F. C. Paratcha G. Ledda F. (2013). Pea3 transcription factor family members Etv4 and Etv5 mediate retrograde signaling and axonal growth of DRG sensory neurons in response to NGF. *J. Neurosci.* 33 15940–15951. 10.1523/jneurosci.0928-13.2013 24089499 PMC6618483

[B17] Forough R. Weylie B. Collins C. Parker J. L. Zhu J. Barhoumi R. (2006). Transcription factor Ets-1 regulates fibroblast growth factor-1-mediated angiogenesis in vivo: role of Ets-1 in the regulation of the PI3K/AKT/MMP-1 pathway. *J. Vasc. Res* 43 327–337. 10.1159/000093198 16682805

[B18] Guo B. Sharrocks A. D. (2009). Extracellular signal-regulated kinase mitogen-activated protein kinase signaling initiates a dynamic interplay between sumoylation and ubiquitination to regulate the activity of the transcriptional activator PEA3. *Mol. Cell Biol.* 29 3204–3218. 10.1128/mcb.01128-08 19307308 PMC2682013

[B19] Harfouche R. Gratton J. P. Yancopoulos G. D. Hussain S. N. A. (2003). Angiopoietin-1 activates both anti- and pro-apoptotic mitogen activated protein kinases. *FASEB J.* 17 1523–1525. 12824293 10.1096/fj.02-0698fje

[B20] Harfouche R. Hassessian H. M. Guo Y. Faivre V. Srikant C. B. Yancopoulos G. D. (2002). Mechanisms which mediate the anti-apoptotic effects of angiopoietin-1 on endothelial cells. *Microvasc. Res.* 64 135–147. 10.1006/mvre.2002.2421 12074640

[B21] Heo S. H. Choi Y. J. Ryoo H. M. Cho J. Y. (2010). Expression profiling of ETS and MMP factors in VEGF-activated endothelial cells: role of MMP-10 in VEGF-induced angiogenesis. *J. Cell. Physiol.* 224 734–742. 10.1002/jcp.22175 20432469

[B22] Hoshino R. Chatani Y. Yamori T. Tsuruo T. Oka H. Yoshida O. (1999). Constitutive activation of the 41-/43-kDa mitogen-activated protein kinase signaling pathway in human tumors. *Oncogene* 18 813–822. 10.1038/sj.onc.1202367 9989833

[B23] Huang D. Ding Y. Luo W. M. Bender S. Qian C. N. Kort E. (2008). Inhibition of MAPK kinase signaling pathways suppressed renal cell carcinoma growth and angiogenesis in vivo. *Cancer Res.* 68 81–88. 10.1158/0008-5472.can-07-5311 18172299

[B24] Iwasaka C. Tanaka K. Abe M. Sato Y. (1996). Ets-1 regulates angiogenesis by inducing the expression of urokinase-type plasminogen activator and matrix metalloproteinase-1 and the migration of vascular endothelial cells. *J. Cell. Physiol.* 169 522–531. 10.1002/(sici)1097-4652(199612)169:3<522::aid-jcp12>3.0.co;2-7 8952701

[B25] Jiwaji M. Daly R. Gibriel A. Barkess G. McLean P. Yang J. (2012). Unique reporter-based sensor platforms to monitor signalling in cells. *PLoS One* 7:e50521. 10.1371/journal.pone.0050521 23209767 PMC3510088

[B26] Kim I. Kim H. G. So J. N. Kim J. H. Kwak H. J. Koh Y. (2000). Angiopoietin-1 regulates endothelial cell survival through the phosphatidylinsitol 3’-kinase/AKT signal transduction pathway. *Cir. Res.* 86 24–29. 10.1161/01.res.86.1.24 10625301

[B27] Kim M. S. Lee C. S. Hur J. Cho H. J. Jun S. I. Kim T. Y. (2009). Priming with angiopoietin-1 augments the vasculogenic potential of the peripheral blood stem cells mobilized with granulocyte colony-stimulating factor through a novel Tie2/Ets-1 pathway. *Circulation* 120 2240–2250. 10.1161/circulationaha.109.856815 19917886

[B28] Kitange G. Shibata S. Tokunaga Y. Yagi N. Yasunaga A. Kishikawa M. (1999). Ets-1 transcription factor-mediated urokinase-type plasminogen activator expression and invasion in glioma cells stimulated by serum and basic fibroblast growth factors. *Lab. Invest.* 79 407–416. 10211993

[B29] Kolizek T. I. Weiss C. Yancopoulos G. D. Deutsch U. Risau W. (1998). Angiopoietin-1 induces sprouting angiogeneis in vitro. *Curr. Biol.* 8 529–532. 10.1016/s0960-9822(98)70205-2 9560344

[B30] Kuma Y. Sabio G. Bain J. Shpiro N. Márquez R. Cuenda A. (2005). BIRB796 inhibits all p38 MAPK isoforms in vitro and in vivo. *J. Biol. Chem.* 280 19472–19479. 10.1074/jbc.m414221200 15755732

[B31] Lee S. H. Bahn J. H. Choi C. K. Whitlock N. C. English A. E. Safe S. (2008). ESE-1/EGR-1 pathway plays a role in tolfenamic acid-induced apoptosis in colorectal cancer cells. *Mol. Cancer Ther.* 7 3739–3750. 10.1158/1535-7163.mct-08-0548 19074849 PMC2643071

[B32] Li R. Pei H. Watson D. K. (2000). Regulation of Ets function by protein - protein interactions. *Oncogene* 19 6514–6523. 10.1038/sj.onc.1204035 11175367

[B33] Lo I. C. Lin T. M. Chou L. H. Liu S. L. Wu L. W. Shi G. Y. (2009). Ets-1 mediates platelet-derived growth factor-BB-induced thrombomodulin expression in human vascular smooth muscle cells. *Cardiovasc. Res.* 81 771–779. 10.1093/cvr/cvn351 19091791

[B34] Matys V. Kel-Margoulis O. V. Fricke E. Liebich I. Land S. Barre-Dirrie A. (2006). TRANSFAC and its module TRANSCompel: transcriptional gene regulation in eukaryotes. *Nucleic Acids Res.* 34 D108–D110. 16381825 10.1093/nar/gkj143 PMC1347505

[B35] Mesquita D. Barros-Silva J. D. Santos J. Skotheim R. I. Lothe R. A. Paulo P. (2015). Specific and redundant activities of ETV1 and ETV4 in prostate cancer aggressiveness revealed by co-overexpression cellular contexts. *Oncotarget* 6 5217–5236. 10.18632/oncotarget.2847 25595908 PMC4467144

[B36] Mofarrahi M. Hussain S. N. (2011). Expression and functional roles of angiopoietin-2 in skeletal muscles. *PLoS One* 6:e22882. 10.1371/journal.pone.0022882 21829546 PMC3146511

[B37] Montuori N. Ragno P. (2014). Role of uPA/uPAR in the modulation of angiogenesis. *Chem. Immunol. Allergy* 99 105–122. 10.1159/000353310 24217605

[B38] Mousavizadeh R. Scott A. Lu A. Ardekani G. S. Behzad H. Lundgreen K. (2016). Angiopoietin-like 4 promotes angiogenesis in the tendon and is increased in cyclically loaded tendon fibroblasts. *J. Physiol.* 594 2971–2983. 10.1113/jp271752 26670924 PMC4887665

[B39] Nakano T. Abe M. Tanaka K. Shineha R. Satomi S. Sato Y. (2000). Angiogenesis inhibition by transdominant mutant Ets-1. *J. Cell. Physiol.* 184 255–262. 10.1002/1097-4652(200008)184:2<255::aid-jcp14>3.0.co;2-j 10867651

[B40] Nwaozuzu O. M. Sellers L. A. Barrand M. A. (2003). Signalling pathways influencing basal and H(2)O(2)-induced P-glycoprotein expression in endothelial cells derived from the blood-brain barrier. *J. Neurochem.* 87 1043–1051. 10.1046/j.1471-4159.2003.02061.x 14622133

[B41] O’Hagan R. C. Tozer R. G. Symons M. McCormick F. Hassell J. A. (1996). The activity of the Ets transcription factor PEA3 is regulated by two distinct MAPK cascades. *Oncogene* 13 1323–1333. 8808707

[B42] Oka T. Rairkar A. Chen J. H. (1991). Structural and functional analysis of the regulatory sequences of the ets-1 gene. *Oncogene* 6 2077–2083. 1945412

[B43] Petrovic N. Bhagwat S. V. Ratzan W. J. Ostrowski M. C. Shapiro L. H. (2003). CD13/APN transcription is induced by RAS/MAPK-mediated phosphorylation of Ets-2 in activated endothelial cells. *J. Biol. Chem.* 278 49358–49368. 10.1074/jbc.m308071200 14507917

[B44] Portales-Casamar E. Thongjuea S. Kwon A. T. Arenillas D. Zhao X. Valen E. (2010). JASPAR 2010: the greatly expanded open-access database of transcription factor binding profiles. *Nucleic Acids Res.* 38 D105–D110. 19906716 10.1093/nar/gkp950 PMC2808906

[B45] Randi A. M. Sperone A. Dryden N. H. Birdsey G. M. (2009). Regulation of angiogenesis by ETS transcription factors. *Biochem. Soc. Trans.* 37 1248–1253. 10.1042/bst0371248 19909256

[B46] Sako K. Fukuhara S. Minami T. Hamakubo T. Song H. Kodama T. (2008). Angiopoietin-1 induces Kruppel-like factor 2 expression through a phosphoinositide 3-kinase/AKT-dependent activation of myocyte enhancer factor 2. *J. Biol. Chem.* 284 5592–5601. 10.1074/jbc.m806928200 19106103

[B47] Sato Y. (2001). Role of ETS family transcription factors in vascular development and angiogenesis. *Cell Struct. Funct.* 26 19–24. 10.1247/csf.26.19 11345500

[B48] Sato Y. Teruyama K. Nakano T. Oda N. Abe M. Tanaka K. (2001). Role of transcription factors in angiogenesis: Ets-1 promotes angiogenesis as well as endothelial apoptosis. *Ann. N. Y. Acad. Sci.* 947 117–123. 10.1111/j.1749-6632.2001.tb03934.x 11795259

[B49] Selvaraj N. Kedage V. Hollenhorst P. C. (2015). Comparison of MAPK specificity across the ETS transcription factor family identifies a high-affinity ERK interaction required for ERG function in prostate cells. *Cell Commun. Signal.* 13:12. 10.1186/s12964-015-0089-7 25885538 PMC4338625

[B50] Shah A. V. Birdsey G. M. Peghaire C. Pitulescu M. E. Dufton N. P. Yang Y. (2017). The endothelial transcription factor ERG mediates Angiopoietin-1-dependent control of Notch signalling and vascular stability. *Nat. Commun.* 8:16002. 10.1038/ncomms16002 PMC5508205 28695891

[B51] Shibuya M. (2006). Differential roles of vascular endothelial growth factor receptor-1 and receptor-2 in angiogenesis. *J. Biochem. Mol. Biol.* 39 469–478. 10.5483/bmbrep.2006.39.5.469 17002866

[B52] Shimizu S. Kageyama M. Yasuda M. Sasaki D. Naito S. Yamamoto T. (2004). Stimulation of in vitro angiogenesis by nitric oxide through the induction of transcription factor ETS-1. *Int. J. Biochem Cell Biol.* 36 114–122. 10.1016/s1357-2725(03)00170-5 14592537

[B53] Siepel A. Bejerano G. Pedersen J. S. Hinrichs A. S. Hou M. Rosenbloom K. (2005). Evolutionarily conserved elements in vertebrate, insect, worm, and yeast genomes. *Genome Res.* 15 1034–1050. 10.1101/gr.3715005 16024819 PMC1182216

[B54] Sivaraman V. S. Wang H. Nuovo G. J. Malbon C. C. (1997). Hyperexpression of mitogen-activated protein kinase in human breast cancer. *J. Clin. Invest.* 99 1478–1483. 10.1172/jci119309 9119990 PMC507966

[B55] Sizemore G. M. Pitarresi J. R. Balakrishnan S. Ostrowski M. C. (2017). The ETS family of oncogenic transcription factors in solid tumours. *Nat. Rev. Cancer* 17 337–351. 10.1038/nrc.2017.20 28450705

[B56] Teruyama K. Abe M. Nakano T. Iwasaka-Yagi C. Takahashi S. Yamada S. (2001). Role of transcription factor Ets-1 in the apoptosis of human vascular endothelial cells. *J. Cell. Physiol.* 188 243–252. 10.1002/jcp.1112 11424091

[B57] Valter M. M. Hügel A. Huang H. J. Cavenee W. K. Wiestler O. D. Pietsch T. (1999). Expression of the Ets-1 transcription factor in human astrocytomas is associated with Fms-like tyrosine kinase-1 (Flt-1)/vascular endothelial growth factor receptor-1 synthesis and neoangiogenesis. *Cancer Res.* 59 5608–5614. 10554042

[B58] Wang J. Huang Q. Liu Z. P. Wang Y. Wu L. Y. Chen L. (2011). NOA: a novel Network Ontology Analysis method. *Nucleic Acids Res.* 39:e87. 10.1093/nar/gkr251 21543451 PMC3141273

[B59] Watanabe D. Takagi H. Suzuma K. Suzuma I. Oh H. Ohashi H. (2004). Transcription factor Ets-1 mediates ischemia- and vascular endothelial growth factor-dependent retinal neovascularization. *Am. J. Pathol.* 164 1827–1835. 10.1016/s0002-9440(10)63741-8 15111329 PMC1615660

[B60] Westermarck J. Li S. P. Kallunki T. Han J. Kahari V. M. (2001). p38 mitogen-activated protein kinase-dependent activation of protein phosphatases 1 and 2A inhibits MEK1 and MEK2 activity and collagenase 1 (MMP-1) gene expression. *Mol. Cell. Biol.* 21 2373–2383. 10.1128/mcb.21.7.2373-2383.2001 11259586 PMC86870

[B61] Witzenbichler B. Maisonpierre P. C. Jones P. Yancopoulos G. D. Isner J. M. (1998). Chemotactic properities of angiopoietin-1 and -2, ligands for the endothelial-specific tyrosine kinase Tie2. *J. Biol Chem.* 273 18514–18521. 10.1074/jbc.273.29.18514 9660821

[B62] Yang S. H. Whitmarsh A. J. Davis R. J. Sharrocks A. D. (1998). Differential targeting of MAP kinases to the ETS-domain transcription factor Elk-1. *EMBO J.* 17 1740–1749. 10.1093/emboj/17.6.1740 9501095 PMC1170521

