# Supplementary material for: ETS1, ELK1, and ETV4 Transcription Factors Regulate Angiopoietin-1 Signaling and the Angiogenic Response in Endothelial Cells
Source: Front Physiol. 2021 Jul 26;12:683651. doi: 10.3389/fphys.2021.683651 (PMC8350579; doi:10.3389/fphys.2021.683651)
Supplement: Supplementary file 1 [file Data_Sheet_1.PDF]

Supplementary Materials Table 1: List of primers used for qPCR measurements.

Type	Sequence	Accession No.	Expected size (bp)
β-ACTIN	F: AGAAAATCTGGCACCACACC<br>R: GGGGTGTTGAAGGTCTCAAA	NM_001101	126
ANGPTL4	F: CCACTTGGGACCAGGATCAC<br>R: CGGAAGTACTGGCCGTTGAG	NM_139314	115
BHLHB2	F: AGAGACGTGACCGGATTAAC<br>R: GAACCACTGCTTTTTCCAAG	NM_003670	102
CDC42EP2	F: ACCAAGGTGCCCATCTATCTG<br>R: CCACTGCCAATATGAATGGTGTG	NM_006779	128
DIPA	F: CTCATGCAGGAGGTGAATCG<br>R: AGTCCAGGAAGCAGCAGAGG	NM_006848	124
DUSP4	F: AGCATCATCTCGCCCAACTT<br>R: AGACCGGAAAGCTGAAGACG	NM_001394	169
DUSP5	F: GGATCCCTGTGGAAGACAGC<br>R: CCACTGCCAATATGAATGGTGTG	NM_004419	221
EGR1	F: CACGCCGAACACTGACATTT<br>R: TAGTCGGGGATCATGGGAAC	NM_001964	135
ELK1	F: ATCTGTGACGCTGTGGCAGT<br>R: CCAGCTTGAATTCACCACCA	NM_001114123	100
ETS1	F: CAAGCCTGTCATTCCTGCTG<br>R: TGAATTCCCAGCCATCTCCT	NM_005238	140
ETV4	F: CCTGAGATCCTCTGGCACCT<br>R: CCCCTCCCTGAGATGTGAAG	NM_001079675	116
FLT1	F: CACTGGGCAGCAGACAAATC<br>R: TCACACCTTGCTTCGGAATG	NM_2019	109
HK2	F: TTTGACCACATTGCCGAATGC<br>R: GGTCCATGAGACCAGGAAACT	NM_000189	117
HMGA2	F: ACCCAGGGGAAGACCCAAA<br>R: CCTCTTGGCCGTTTTTCTCCA	NM003483	92

KLF2	F: GCACGCACACAGGTGAGAAG<br>R: ACCAGTCACAGTTTGGGAGGG	NM_016270.2	269
PLAU	F: GTGAGCGACTCCAAAGGCA<br>R: GCAGTTGCACCAGTGAATGTT	NM_025195	126
RAPGEF5	F: AAGACCTGGCGGACACTTTG<br>R: GCTCTTGCTCGTGAATTGAATTG	NM_012294	142
STC1	F: TGGTGATCAGTGCTTCTGCAAC<br>R: CTCAGTGATGGCTTCAGGGTTC	NM_003155	169
TRIB1	F: GAGGAGAGAACCCAGCTTAGA<br>R: GAGGATCTCAGGGCTCACGTA	NM_025195	179
VEGF	F: CTACCTCCACCATGCCAAGT<br>R: CACACAGGATGGCTTGAAGA	NM_001025366	281

Supplementary Materials Table 2: Description of luciferase reporters.

Plasmid	TF	Sequence	Repeats	Sequence ID	Reference	Unique reporter sequence	Specific F qPCR primer sequence
pMN191	ELK1	CCATGGAGGG		T00250	[1-2]	CCCGCGTGCCCGTG TTCCTTTTT	GTGCCCGTGTTCCTTT
pMN141	ETS1	GGAGGAAGT	3x	R04051	[3]	GCGCTCCCTCTGTT GCGCTCCCT	CTCTGTTGCGCTCCCT

References:

1. Messeguer X, Escudero R, Farre D, Nunez O, Martinez J, Alba MM (2002) PROMO: detection of known transcription regulatory elements using species-tailored searches. *Bioinformatics* 18: 333-334.

2. Farre D, Roset R, Huerta M, Adsuara JE, Rosello L, Alba MM, Messeguer X (2003) Identification of patterns in biological sequences at the ALGGEN server: PROMO and MALGEN. *Nucleic Acids Res* 31: 3651-3653.

3. Romanov S, Medvedev A, Gambarian M, Poltoratskaya N, Moeser M, Medvedeva L, Gambarian M, Diatchenko L, Makarov S (2008) Homogenous reporter system enables quantitative functional assessment of multiple transcription factors. *Nature Methods* 5: 253-260.

Supplementary Materials Table 3: Z-scores for Ang-1 dataset over TRANSFAC profiles (ETS-type family).

Matrix	Transcription Factor	Z-score
M00025	ELK-1	6.97
M00771	ETS-1	7.43
M00655	ETV-4	6.15
M00340	c-ETS-2	4.90
M00497	STAT3	1.94
M00499	STAT5A	1.70
M00175	AP-4	1.57

Supplementary Materials Table 4: Enrichment in biological process for fifteen gene subset upregulated by Ang-1.

GO:Term	Term Name	No. of genes
GO:0030334	Regulation of cell migration	7
GO:0001525	Angiogenesis	6
GO:00512703	Regulation of cellular component movement	7
GO:0040012	Regulation of locomotion	7
GO:0043536	Positive regulation of blood vessel endothelial cell migration	3
GO:0030947	Regulation of VEGFR signaling pathway	3
GO:00422211	Response to chemical stimulus	11
GO:0001666	Response to Hypoxia	5
GO:00704823	Response to oxygen levels	5
GO:00435354	Regulation of blood vessel endothelial cell migration	3

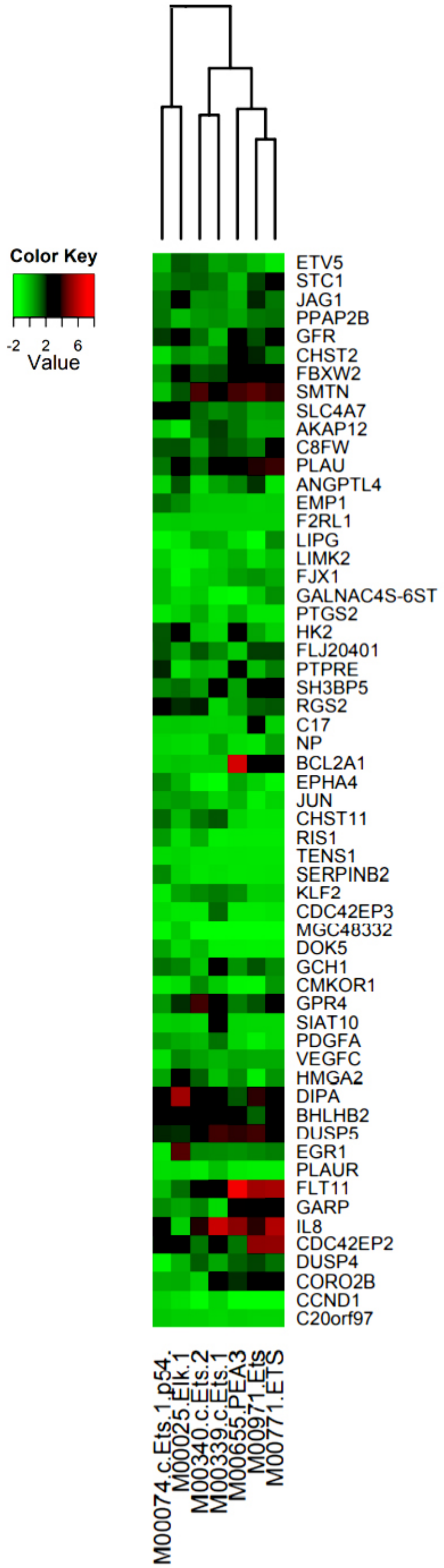

Supplementary Figure 1: Predicted ETS TF regulators of Ang-1-induced genes in ECs. Heat map representing 58 genes (Y axis) that were upregulated in HUVECs exposed for 4 h to Ang-1. Genes were clustered according to Z-scores obtained from sixty-two PWMs generated using TRANSFAC and JSAPAR

databases and based on flanking regions of 10 kb upstream or downstream of each gene. Color scale represents value of Z-score.

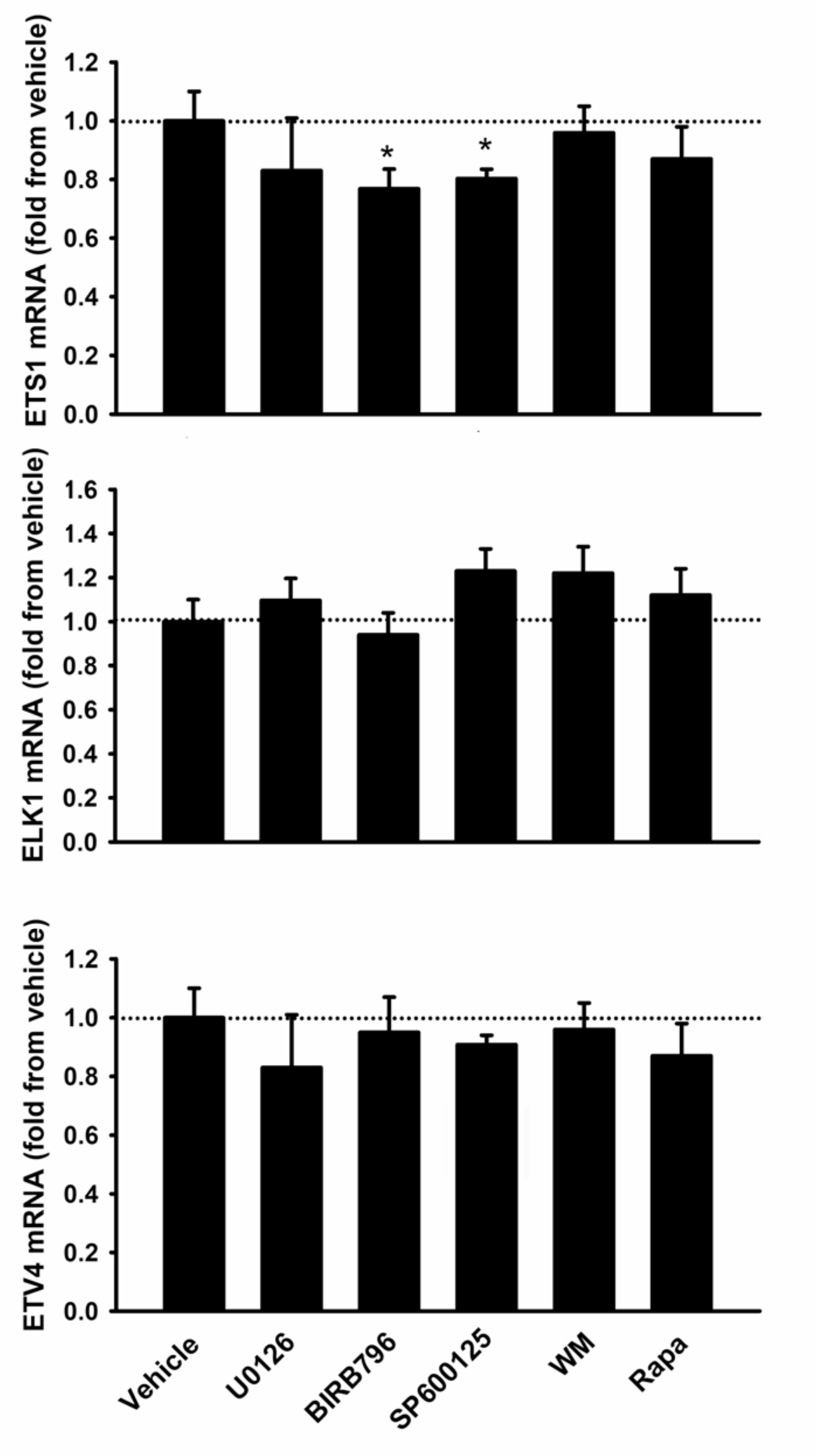

Supplementary Figure 2: mRNA levels of ETS1, ELK1, and ETV4 in HUVECs pre-incubated for 1 h with various pathway inhibitors and then exposed to PBS. *P<0.05, compared to vehicle. N=6 per.

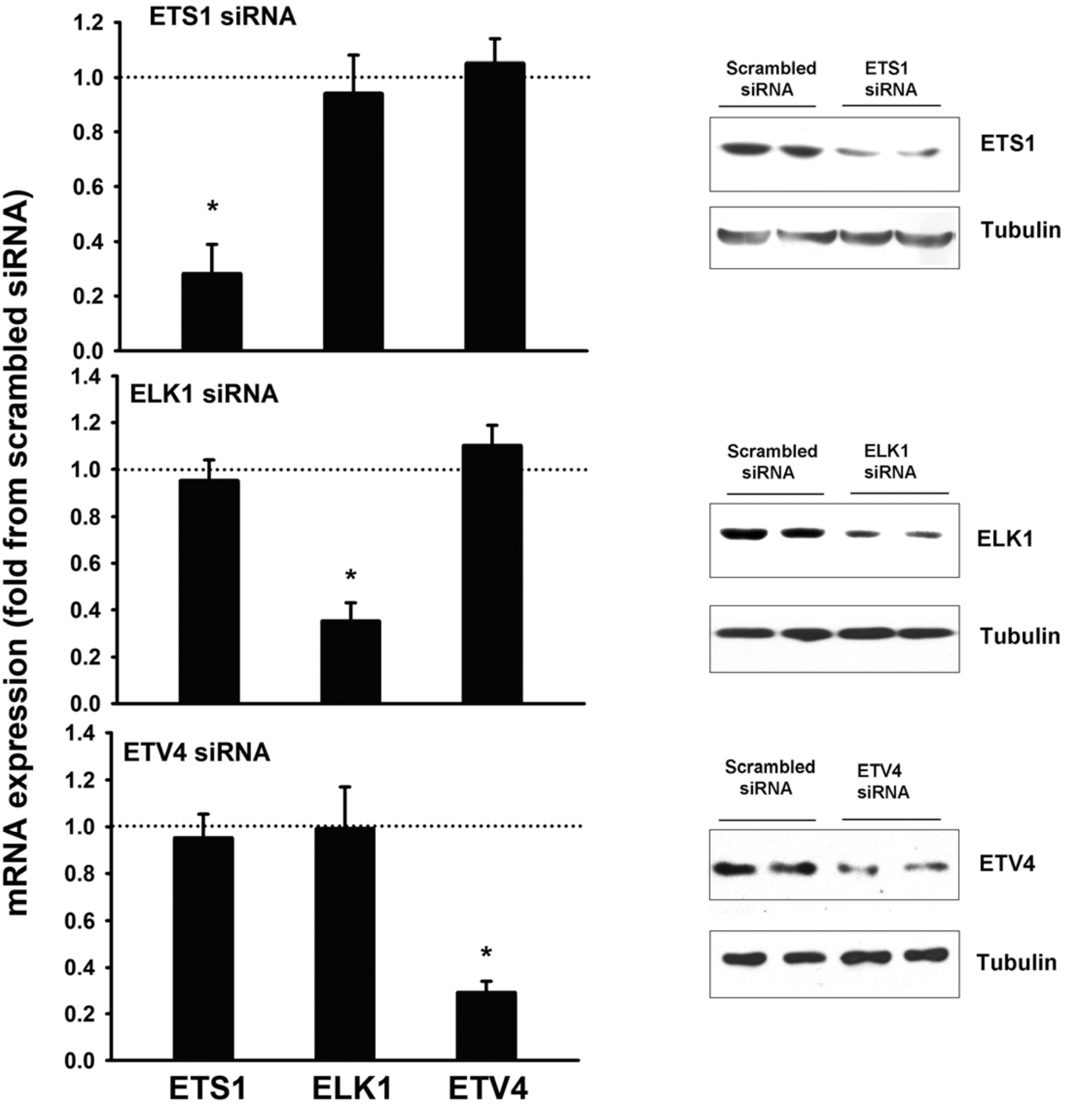

Supplementary Figure 3: Effects of selective siRNA oligos on ETS1, ELK1, and ETV4 mRNA and protein levels in HUVECs. Results of mRNA are mean ±SEM and expressed as fold change from values measured in cells transfected with scrambled siRNA oligos. *p<0.05, compared to scrambled siRNA.

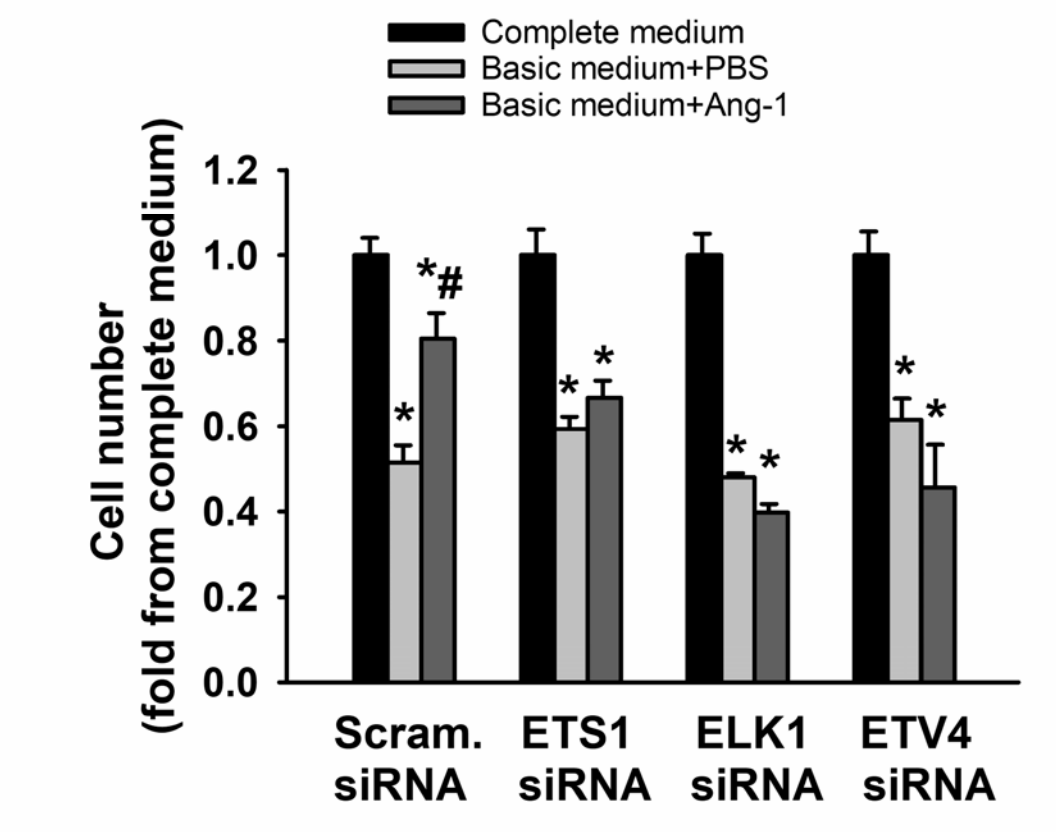

Supplementary Figure 4: ETS1, ELK1, and ETV4 contribute to Ang-1-induced EC survival. Cell counts of HUVECs transfected with scrambled, ETS1, ELK1, or ETV4 siRNA oligos. Equal numbers of cells were maintained in complete (20% FBS), basic medium (2% FBS) containing PBS, or Ang-1 (300 ng/ml). Cells counted 24 h later. Values are means ± SEM and expressed as fold from control medium. *P<.05, compared to complete medium. [#]P<.05, compared to PBS.
